# Microwave ablation combined with transcatheter arterial chemoembolization is effective for treating unresectable hepatoblastoma in infants and children

**DOI:** 10.1097/MD.0000000000012607

**Published:** 2018-10-19

**Authors:** Yizhou Jiang, Shaoyi Zhou, Gang Shen, Hua Jiang, Jing Zhang

**Affiliations:** Department of Interventional Radiology and Vascular Anomalies, Guangzhou Women and Children's Medical Center, Jinan University, Guangzhou, P.R. China.

**Keywords:** children, hepatoblastoma, microwave ablation, transcatheter arterial chemoembolization

## Abstract

The present study is to evaluate the feasibility and efficacy of microwave ablation (MWA) combined with transcatheter arterial chemoembolization (TACE) in the treatment for unresectable hepatoblastoma in infants and children. A total of 17 patients with PRETEXT stage III and IV hepatoblastoma that was unresectable by conventional resection were included in the present study. The patients were treated with TACE, MWA, and chemotherapy. All cases were diagnosed by computed tomography (CT) and liver tumor biopsy before TACE procedure. All patients received 2 courses of TACE and 1 to 2 times of MWA. Finally, several cycles of chemotherapy were arranged. Among the 17 patients, 14 were alive and had normal alpha-fetoprotein (AFP) levels. The other 3 patients died from tumor progression. The follow-up periods ranged from 10 to 68 months. Complete ablation was achieved in the 14 patients (14/17, 82.35%). Most patients were well tolerated during the whole course except for 1 patient with pneumonedema after TACE and another 1 with biloma after MWA. No marked chemotherapeutic agent-induced toxicity occurred. After chemotherapy or TACE, transient blood indicators and symptoms were observed as follows: myelosuppression, abnormal liver function, gross hematuria, fever, and abdominal pain. Transient symptoms after MWA were fever, abdominal pain, and massive gross hematuria. The present study demonstrates that MWA combined with TACE is a safe and effective method for treating unresectable hepatoblastoma in infants and children with controllable side effects.

## 1 Introduction

Hepatoblastoma is the most common liver tumor of early childhood and two-thirds of hepatic malignancies in children are hepatoblastomas.^[1]^ It is well established that effective treatment of hepatoblastoma requires complete resection. With complete surgical resection and chemotherapy, the prognosis of the disease can be significantly improved.^[2]^ However, 30% of patients with PRETEXT stage III and IV hepatoblastoma^[3]^ still can’t undergo operation after chemoreduction treatment due to extensive multifocality, or extensive hepatic venous or portal venous involvement. Liver transplantation (LT) seems to be another accepted method for these patients,^[4,5]^ but it is not widely used in China because of low rate of organ donation. Although long-term outcome in pediatric LT is excellent, the side effects of immunosuppression cannot be denied.^[6,7]^ Microwave ablation (MWA) is a safe and effective technology that is widely used in adults for the treatment of both benign and malignant solid lesions.^[8–10]^ However, its use in the treatment of hepatoblastoma has not been reported yet. As a mature therapy, transcatheter arterial chemoembolization (TACE) has already been applied to the treatment for hepatoblastoma. Based on the above, we decide to apply MWA in combination with TACE on patients with stage III and IV hepatoblastoma. The subset of patients who remain unresectable by standard resection after TACE are the subjects of the present study.

## 2 Materials and methods

### 2.1 Patients

A total of 17 patients with PRETEXT III and IV tumors treated between July 2010 and January 2014 were retrospectively reviewed, including 10 boys and 7 girls. The age of the patients ranged from 4 months to 56 months, with a median age of 13 months. All cases were diagnosed by computed tomography (CT), alpha-fetoprotein (AFP), and liver tumor biopsy before TACE procedure. According to PRETEXT system, there were 6 cases at stage III and 11 cases at stage IV. All procedures were approved by the Ethical Committee of Guangzhou Women and Children's Medical Center. Written informed consents were obtained from legal guardians of all patients.

None of the patients received any treatment before hospitalization in our center. After hospitalization, plain chest CT scans and plain and contrast-enhanced upper abdomen CT scans were performed in all cases. Moreover, essential blood tests were performed, including AFP, routine blood test, biochemical tests, etc. All patients were not suitable for surgical resection according to experienced oncologists and surgeons when TACE was finished. TACE and MWA were performed by the same team of doctors.

### 2.2 Transcatheter arterial chemoembolization

A catheter is placed in the hepatic artery and its branches were selected. Under general anesthesia, the right/left femoral artery was catheterized using Seldinger technique. Percutaneous liver biopsy would be performed under B-ultrasound guidance before TACE. Then, an intra-arterial catheter (4F, Terumo, Tokyo, Japan) was percutaneously inserted under the condition of whole body heparinization. The dose of heparin (Wanbang Pharmaceutical Factory, Jiangsu, China) was 100 iu/kg bodyweight (75 iu/kg if bodyweight was less than 10 kg). Under fluoroscopic digital subtraction angiography (DSA), a 4F Cobra catheter was manipulated into the celiac trunk. After celiac trunk arteriography, a 2.7F micro-catheter (4F, Terumo, Tokyo, Japan) was selectively catheterized to tumor-feeding artery. Hepatoblastoma was mainly supplied by celiac trunk, but sometimes the nourishing vessels were composed of renal artery, lumbar artery, internal thoracic artery, etc. Therefore, the ”integrity” of the tumor should be carefully checked by comparing angiography with CT scan. When tumor feeding artery was confirmed, interventional chemotherapy drugs were injected into the artery. Routine intra-operative medication was Cisplatin (60 mg/m^2^, Qilu Pharmaceutical Factory, Jinan, China) and Pirarubicin (30 mg/m^2^, Main Luck Pharmaceuticals, Shenzhen, China). Standard doses of Cisplatin and Doxorubicin were mixed with Lipiodol (8–10 mL, Laboratoire Guerbet, Roissy Charles de Gaulle, France) and contrast agent (5 mL, GE Healthcare, Shanghai, China). After that, microspheric granules (100–300 μm, BioSphere Medical, S.A., Paris, France) were injected to increase the density of embolism. In order to evaluate curative effect and to sustain best operation results, routine CT scan, AFP, and other hematologic examinations were performed before the next TACE 4 weeks later.

### 2.3 Microwave ablation

MWA was performed under general anesthesia 2 weeks after TACE. The microwave equipment used in the present study was Nanjing Qing Hai (FORSEA) type MTC-1/2/3, with a frequency of 2450 MHz, an adjustable output power of 10 to 120 W and a cooled microwave needle of 14 G/150 cm or 16 G/150 cm. During CT-guided thermoablation, the presence of Lipiodol (deposited during TACE) helped properly visualize and delineate the target tumor. We used the 16 G antenna with a power output of 60 to 70 W and the duration of energy delivery was 5 minutes. During MWA operation, MWA probe could reach the lesions accurately and avoid great vessels and important organs with the help of Ig4 navigation system (Veran Medical Technologies Inc, St. Louis, MO). The Ig4 navigation system shared real-time CT images and provided real-time positioning information. Plans of ablation were made immediately after CT scans were finished. The puncture angle of probe, ablation area, and number of skin punctures were considered. When withdrawing the antenna, the needle tracks were routinely cauterized to avoid tumor seeding and bleeding. A month later, patient needed a second MWA ablation if lesions were incompletely ablated according to CT scan.

### 2.4 Chemotherapy

When TACE and MWA were finished, several cycles of chemotherapy were arranged for avoiding distant metastases. Patients with PRETEXT IV tumor were treated according to SIOPEL-3HR study.^[11]^ Each patient received 4 cycles of this regimen (cisplatin: 80 mg/m^2^/dose D1; carboplatin: 500 mg/m^2^/dose D14; doxorubicin: 60 mg/ m^2^/dose D14; interval was 14 days). Patients with PRETEXT III tumor were also given 4 cycles of chemotherapy by C5 V regimen (cisplatin: 100 mg/m^2^/dose D1; 5-fluorouracil: 600 mg/m^2^/dose D3; vincristine: 1.5 mg/m^2^/dose D3, D10, D17).^[12]^

### 2.5 Statistical analysis

SPSS 16.0 software (IBM, Armonk, NY) was used to perform statistical analyses for the present study. Difference with *P* < .05 was considered statistically significant. Continuous data were expressed as means ± standard deviations (SD). A paired sample *t* test was used for comparing tumor diameter and AFP values before and after treatment.

## 3 Results

### 3.1 General therapeutic conditions

Among 17 patients, 14 were alive and their AFP results in follow-ups were normal. However, the other 3 patients died from tumor progression with portal vein embolus. All patients completed the whole course of treatment, and received 2 times of TACE. In addition, 11 patients underwent 1 time of MWA and the other 6 patients received 2 times of MWA. After TACE and MWA, 4 cycles of chemotherapy were given to all patients. The most common complication of TACE and MWA was abnormal liver function, in which alanine aminotransferase level was elevated.

### 3.2 Transcatheter arterial chemoembolization

After TACE, especially within 5 days after operation, all patients had fever, upper abdominal pain, nausea, and vomiting. Their body temperature ranged from 37.5°C to 40.3°C, and lasted for 3 to 16 days. Fever and upper abdominal pain recovered well after suitable treatment, and nausea and vomiting were transient. Of note, 6 patients had postoperative hemoglobin urine and 10 patients suffered from poor appetite during the whole course of TACE. No marked chemotherapeutic agent-induced toxicity was noted during TACE. However, 1 patient got pneumonedema immediately after TACE and received respiratory and circulatory support in pediatric intensive care unit for 5 days. The patient recovered well and was discharged at last.

### 3.3 Microwave ablation

**Figure 1:**
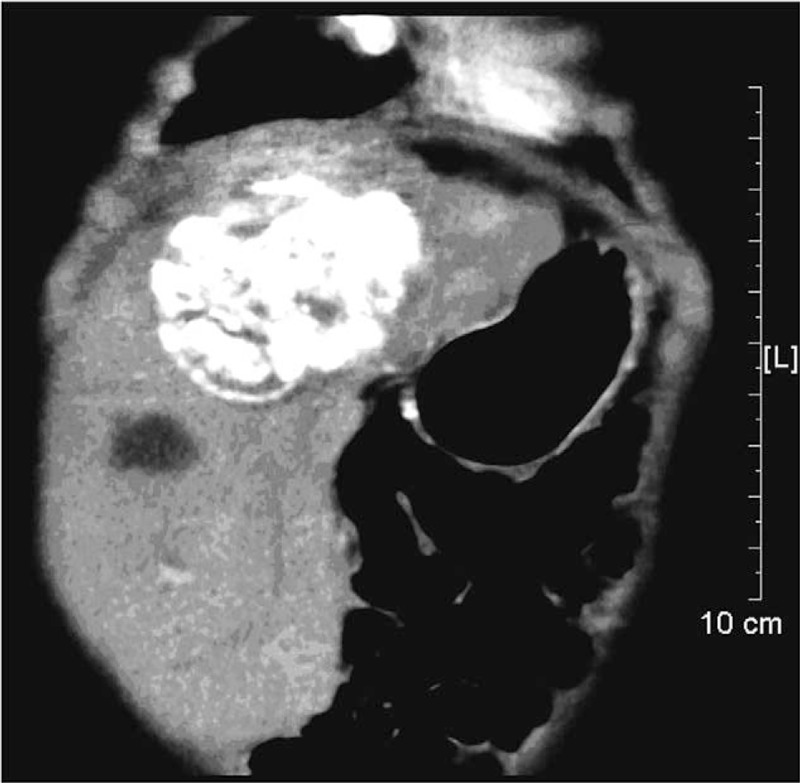
Contrast-enhanced CT scan showing a biloma with fluid collection. CT = computed tomography.

All 17 patients received a total of 23 times of MWA. Complete ablation was achieved in 14 patients (14/17, 82.35%), and AFP level was also decreased to normal level in these patients. Common complications after MWA included hemoglobin urine, upper abdominal pain, and fever. All of these complications were transient or controllable. Hemoglobin urine lasted for 1 to 3 days due to the absorption of necrotic material. The patients were recovered after receiving hydration. All patients tolerated well after the procedures and remained in the hospital for about 3 days. The shapes of liver and gallbladder were normal after MWA. Moreover, there was no active bleeding in liver according to abdominal ultrasound. A biloma in the right lobe of liver was found 1 month after MWA in a patient (No. 5) (Fig. 1). However, the patient didn’t have any clinic symptoms. CT examination on this patient performed 4 months after MWA revealed nearly complete resolution of biloma.

### 3.4 Tumor and AFP responses to treatment and prognosis

**Figure 2:**
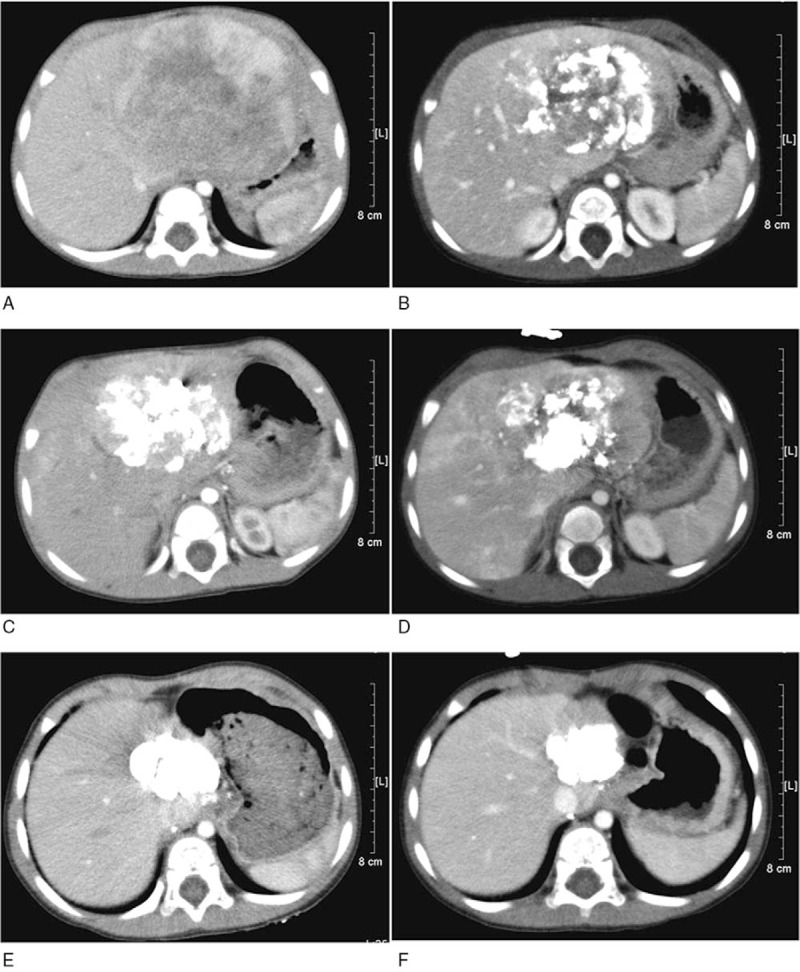
A 1-year-old boy with PRETEXT IV hepatoblastoma who had experienced 2 times of TACE, 1 time of MWA, and 4 cycles of chemotherapy. (A) Contrast-enhanced computed tomography (CT) scan showing a huge lesion in the liver. (B) Contrast-enhanced CT scan revealing that Lipiodol deposited within a part of the tumor after 1 time of TACE was performed. (C) Lipiodol deposited within most parts of the lesion in CT scan after 2 times of TACE. (D) Active lesion necrosis was observed in contrast-enhanced CT scan after MWA. (E) Contrast-enhanced CT scan at 6 months after MWA. The necrotic part of the lesion was absorbed while the other part of the lesion was deposited with high-density Lipiodol. (F) Contrast-enhanced CT at 15 months after MWA revealing that the tumor was absorbed gradually with no active lesion. CT = computed tomography, TACE = transcatheter arterial chemoembolization, MWA = microwave ablation.

After 2 courses of TACE, tumor volumes were reduced at a high level, with a shrinkage between 63.68% and 93.00% (mean, 81.14%; *t* = 6.37; *P* = .000). At the same time, AFP level decreased rapidly with the step of tumor shrinking. The decreasing rate ranged from 35.78% to 99.54% (mean, 83.61%; *t* = 4.636; *P* = .000). After MWA, tumor volumes were further reduced in varying degrees. The shrinkage rate was from 36.41% to 90.05% (mean, 76.11%; *t* = 6.073; *P* = .000) compared with the values before MWA (Table 1). CT images of tumors also showed these changes after treatment (Fig. 2). Meanwhile, AFP levels were decreased gradually. However, AFP levels of 14 patients who got complete ablation were decreased to normal levels, and those of the other 3 patients were still much higher than normal levels. The follow-up periods of the patients ranged from 10 to 68 months. The survival rate of the patients was 82.35% (14/17). The survival time of the 3 died patients was relatively short (10 months, 12 months, and 13 months). Two patients died of portal vein tumor thrombus and 1 patient died of pulmonary metastasis.

**Table 1:**
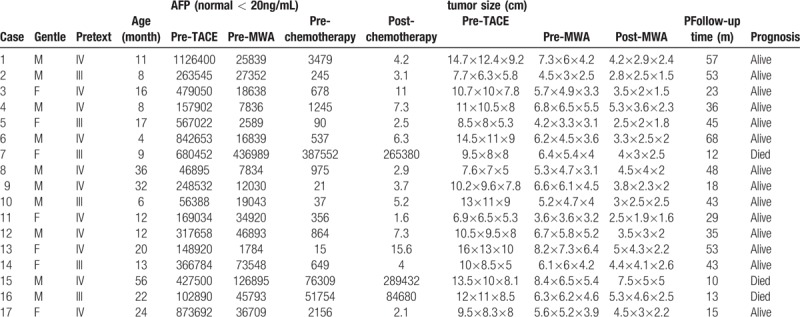
Clinical data of 17 patients.

## 4 Discussion

With an incidence of 0.6 to 1.2 per million, hepatoblastoma is a rare but the most frequent hepatic tumor in children younger than 5 years old.^[13]^ Complete surgical resection is considered to be the key to the treatment for hepatoblastoma.^[2,14]^ However, the tumors of lots of children are too advanced to be resected at the initial stage. In recent years, TACE has shown benefits for patients with large hepatoblastoma.^[15]^ With the help of TACE reduction effect, many children have been treated with TACE firstly to achieve the standard of surgical resection. However, some patients still can’t get perfect resection for a variety of reasons. Therefore, TACE combined with MWA may be an alternative strategy for these patients.

Thermal ablation such as radiofrequency ablation (RFA) and MWA has become a widely used and accepted tool for many solid tumors. However, its use in the treatment for hepatoblastoma has only been described recently. Wang et al have treated 12 patients with unresectable hepatoblastoma by high-intensity focused ultrasound and achieved satisfied outcomes with 10 patients being alive.^[16]^ It is thought that RFA or MWA is suitable for tumors with a diameter less than 3 cm. Recently, Thamtorawat et al^[17]^ have carried out some work for hepatocellular carcinoma (HCC) with a diameter up to 5 cm with MWA, and gained high efficacy. Their outcomes show low complication and high tumor control rates, not only for small tumors but also for intermediate-size tumors between 3 cm and 5 cm.^[17]^ Compared to other approaches, MWA has additional advantages such as higher rate of temperature increase, high thermal efficiency, stable and controllable thermal field, and good hemostasis. Abdelaziz et al^[18]^ have studied TACE and MWA or RFA for HCC. They report that TACE-MWA leads to better response rates than TACE-RFA on tumors with diameters of 3 to 5 cm. Chen et al^[19]^ have used MWA and TACE on HCC and demonstrated that TACE/MWA lead to better responses than TACE on HCC tumors with diameters smaller than 5 cm. Therefore, we chose MWA technique in the present study.

In the present study, tumor volume has a sharp decrease after 2 times of TACE. The shrinkage is from 63.68% to 93.00%. Meanwhile, AFP reduction rate is between 35.78% and 99.54%. As mentioned in the method of TACE, hepatoblastoma nourishing vessels are composed of renal artery, lumbar artery, internal thoracic artery, and so on. Unfortunately, a patient (No. 9) has developed pneumonedema that is caused by a very small hepatic and pulmonary arteriovenous fistula when infusing drugs to the tumor. After a few days in intensive care unit, the patient is recovered and Lipiodol is absorbed by the lungs. This phenomenon has never been reported in previous literatures. Usually, the radiation field we are concerned is only the liver. We suggest that we pay more attention to the lungs during the process of TACE.

In the present study, complete ablation is achieved in 14 patients (14/17, 82.35%), and AFP levels in these patients are also decreased to normal levels. Postoperative complications including hemoglobin urine, upper abdominal pain, and fever are transient. Besides, we have noticed that a patient (No. 5) has formed a biloma in the right lobe of liver a month after MWA. The development of biloma after RFA, MWA, or TACE in adults with HCC is not rare,^[20–22]^ but this is the first time for a child after ablation. The incidence of biloma in our study is 4.35% (1/23, 4.35%). Our finding is similar with the report by Chang et al^[23]^ about biloma formation after RFA of HCC (3.3%, 109/3284). We hypothesize that the formation of biloma is associated with bile duct injury during ablation. The patient has no clinical symptoms, so we have not carried out any treatment about the lesion. In addition, CT examination performed 4 months after MWA reveals nearly complete resolution of biloma. Furthermore, a new technology called Ig4 navigation system (Veran Medical Technologies Inc, St Louis, MO) is used in MWA process. Ig4 navigation system can share real-time CT images. Then, we are able to make plans for ablation immediately after CT scans are finished. Moreover, Ig4 navigation system provides real-time positioning information when we do percutaneous puncture. This technology makes surgery safer, because it avoids punctures to large blood vessels.

Unfortunately, 3 patients have died from tumor progression with portal vein embolus. The overall survival rates from 1 to 4 years are 94.12%, 64.70%, 52.94%, and 29.41%, respectively. To our delight, a patient (No. 6) has survived with tumor for 68 months during follow-up. It means that the combination of MWA with TACE can be a useful and effective method for patients with unresectable hepatoblastoma.

In conclusion, the present study demonstrates that the therapeutic effect of the combination of MWA and TACE for unresectable hepatoblastoma is exciting. The deficiency of this study is that the sample size is not large enough and follow-up time is not long enough. All statistical results will be more reliable if the sample size is larger. Moreover, long-term complications such as local recurrence, distant metastasis, secondary tumors, and whether these will affect the growth and development of children are still unknown. More work should be completed in the future on this study.

## Acknowledgments

We would like to thank Wenchan Xu for help preparing this article.

## Author contributions

**Conceptualization:** Yizhou Jiang, Shaoyi Zhou.

**Data curation:** Yizhou Jiang.

**Formal analysis:** Hua Jiang.

**Investigation:** Shaoyi Zhou, Hua Jiang.

**Methodology:** Shaoyi Zhou, Gang Shen, Hua Jiang.

**Project administration:** Jing Zhang, Shaoyi Zhou, Gang Shen.

**Resources:** Jing Zhang, Shaoyi Zhou, Gang Shen.

**Supervision:** Jing Zhang.

**Validation:** Yizhou Jiang, Gang Shen, Hua Jiang.

**Visualization:** Yizhou Jiang.

**Writing – original draft:** Yizhou Jiang.

**Writing – review & editing:** Yizhou Jiang.
